# The course of health-related quality of life from diagnosis to two years follow-up in patients with oropharyngeal cancer: does HPV status matter?

**DOI:** 10.1007/s00520-020-05932-w

**Published:** 2021-01-17

**Authors:** Laura H. A. Korsten, Femke Jansen, Birgit I. Lissenberg-Witte, Marije Vergeer, Ruud H. Brakenhoff, C. René Leemans, Irma M. Verdonck-de Leeuw

**Affiliations:** 1 Department of Otolaryngology-Head and Neck Surgery, Amsterdam UMC, Vrije Universiteit Amsterdam, P.O. Box 7057, 1007 MB Amsterdam, the Netherlands; 2 Cancer Center Amsterdam (CCA), Amsterdam, Netherlands; 3 Amsterdam Public Health research institute, Amsterdam, Netherlands; 4 Department of Clinical, Neuro- and Developmental Psychology, Amsterdam Public Health research institute, Vrije Universiteit Amsterdam, Amsterdam, the Netherlands; 5 Amsterdam UMC, Vrije Universiteit Amsterdam, Department of Epidemiology and Biostatistics, Amsterdam, the Netherlands; 6 Department of Radiation Oncology, Amsterdam UMC, Vrije Universiteit Amsterdam, Amsterdam, the Netherlands

**Keywords:** Head and neck cancer, Human papilloma virus, HPV, Health-related quality of life, Oropharyngeal cancer, Cohort study

## Abstract

**Purpose:**

To investigate the course of health-related quality of life (HRQOL) from diagnosis to 2 years follow-up among patients with oropharyngeal cancer (OPSCC), in relation to human papilloma virus (HPV) status.

**Methods:**

This study included 270 OPSCC patients. Age, sex, tumor sublocation, tumor stage, HPV status, treatment modality, comorbidity, smoking, and alcohol use were retrieved from medical records. HPV status was positive when p16 and HPV DNA tests were both positive. HRQOL was assessed using the EORTC QLQ-C30/QLQ-H&N35 pretreatment and at 6 weeks, 6, 12, 18, and 24 months after treatment. To compare the course of HRQOL between patients with an HPV-positive versus HPV-negative tumor, linear and logistic mixed models were used.

**Results:**

Patients with an HPV-positive tumor (29%) were more often male, diagnosed with a tumor of the tonsil or base of the tongue, treated with single treatment, had fewer comorbidities, were less often current smokers and had lower alcohol consumption. Adjusted for confounders, the course of global quality of life, physical, role, and social functioning, fatigue, pain, insomnia, and appetite loss was significantly different: patients with an HPV-positive tumor scored better before treatment, worsened during treatment, and recovered better and faster at follow-up, compared to patients with an HPV-negative tumor. The course of emotional functioning and oral pain was also significantly different between the two groups, but with other trajectories.

**Conclusion:**

The course of HRQOL is different in patients with an HPV-positive tumor versus an HPV-negative tumor, adjusted for sociodemographic, clinical, and lifestyle confounders.

## Introduction

Head and neck cancer (HNSCC) is the sixth most common cancer worldwide [1]. The main risk factors for HNC are tobacco use and excessive alcohol consumption [2–4]. Also infection by the human papillomavirus (HPV) has been shown to be a risk factor in developing HNC, especially in oropharyngeal carcinoma (OPSCC) [5, 6]. In clinical practice, patients with an HPV-positive OPSCC respond better to treatment and seem to report better health-related quality of life (HRQOL), compared to patients with an HPV-negative OPSCC. Previous studies showed that HNSCC patients with an HPV-positive OPSCC have a better prognosis and survival rate is higher as compared to patients with an HPV-negative tumor [7–11]. Previous literature reviews on HRQOL in OPSCC patients [12, 13] concluded that subanalyses investigating the association between HRQOL and HPV status were not possible because the inclusion periods of most studies predated regular HPV testing. At present, we found ten studies that investigated HPV status in relation to HRQOL among HNSCC patients [14–23]. Findings from these studies suggest that HRQOL of patients with an HPV-positive tumor (compared to those diagnosed with an HPV-negative tumor) may be better before treatment, deteriorates much more during treatment, and recovers better from three months follow-up and beyond, but this pattern may be confounded by sociodemographic, clinical, and lifestyle factors [17]. Obviously, there is still a need for more research into the association between HPV status and HRQOL in OPSCC patients. Therefore, the aim of the present study was to investigate the course of HRQOL (as measured with the EORTC QLQ-C30 and QLQ-H&N35) among OPSCC patients in relation to HPV status, from pretreatment to 24-month follow-up, taking into account possible sociodemographic and clinical confounders. The results of this study are highly relevant for clinical practice to tailor information and supportive care in a personalized way taking HPV status into account.

## Materials and methods

### Study population

The study population consisted of patients diagnosed with primary squamous cell carcinoma of the mucosal surfaces of the oropharynx (OPSCC) and treated with curative intent between January 1999 and January 2011 at the Amsterdam UMC, location VUmc in Amsterdam. Exclusion criteria were distant metastases, second primary tumors, previous surgery or radiotherapy for HNSCC, brachytherapy, serious cognitive impairment or lack of basic knowledge of the Dutch language. We also excluded patients who did not complete pretreatment HRQOL questionnaires and of whom HPV status was not assessed.

Sociodemographic (age, sex), clinical (comorbidity (none, mild, moderate, severe), tumor sublocation: tonsil, base of tongue, soft palate/uvula, other oropharyngeal locations), TNM tumor stage (stage I – IV according to the American Joint Committee on Cancer 7th edition) [24], HPV status (positive, negative), treatment modality (categorized as single (surgery or radiotherapy) or combination (surgery and (chemo) radiotherapy, or chemoradiation)), and lifestyle (smoking (pack years), and alcohol use (units per year)) variables were retrieved from medical records. Comorbidity was assessed using the Adult Comorbidity Scale-27 (ACE-27), a validated instrument with four grades of comorbidity (none, mild, moderate, or severe) [25]. Tumor biopsies of all patients were tested for HPV according to the validated test algorithm for HPV-detection, consisting of p16^INK4A^ immunostaining followed by high risk HPV DNA-detection on formalin-fixed paraffin-embedded (FFPE) tumor specimen [26]. HPV status was scored as HPV-positive when p16 and HPV DNA tests were both positive. Treatment modality was categorized into single treatment (surgery or radiotherapy alone) versus combination treatment (surgery, radiotherapy, chemotherapy).

HRQOL data was collected as part of standard clinical care. Since 1999 (department of Radiotherapy) and 2016 (department of Otolaryngology-Head and Neck Surgery), HNC patients are asked to complete patient-reported outcomes measures (PROMs) on HRQOL using a paper-and-pencil version of the PROMs and a touch screen computer-assisted data collection system called OncoQuest respectively. Patients are asked to complete these PROMs in the clinic before start of treatment and at every follow-up visit at one of the two departments. Information on durable usage of these PROMs in our clinic can be found elsewhere [27]. All patients were asked for informed consent to use their data for scientific research. Patients were only included in this study when they provided such informed consent. According to the Dutch Medical Research Involving Human Subjects Act, ethical approval was not necessary, because patients were not subjected to procedures or required to follow rules of behavior.

### HRQOL outcome measures

HRQOL was assessed using the EORTC QLQ-C30 and EORTC QLQ-H&N35. The 30-item EORTC QLQ-C30 is a generic HRQOL questionnaire that consists of nine domains and six single items. These nine domains include global quality of life, physical functioning, role functioning, emotional functioning, cognitive functioning, social functioning, fatigue, nausea and vomiting, and pain. Single items encompass: dyspnea, insomnia, appetite loss, constipation, diarrhea, and financial difficulties. The scores of the QLQ-C30 are linearly transformed to a scale of 0–100, with a higher score suggesting a higher (i.e., more positive) level of functioning or global HRQOL, or a higher (i.e., more negative) level of symptoms or problems [28, 29]. The EORTC QLQ-H&N35 is an HNC-specific HRQOL questionnaire that consists of seven scales and eleven single items. The scales include oral pain, swallowing, senses, speech, social eating, social contact, and sexuality. The single items consists of items about problems with teeth, problem with opening the mouth, dry mouth, sticky saliva, coughing, feeling ill, use of painkillers, nutritional supplements, feeding tube, losing weight, or gaining weight. The scores of the QLQ-H&N35 are linearly transformed to a scale of 0–100, with a higher score suggesting a higher (i.e., more negative) level of symptoms or problems [29]. Patients were asked to complete the questionnaires at baseline (pretreatment) and at 6 weeks and 6, 12, 18, and 24 months after treatment.

### Statistical analyses

Descriptive statistics were generated for the range of patient characteristics and outcome variables. Chi-square tests were used to examine differences between patients with an HPV-positive versus HPV-negative tumor, with respect to sex, comorbidity, tumor sublocation, tumor stage, treatment modality, smoking, and alcohol use. Age was compared with the independent samples *t* test. To compare the longitudinal course of HRQOL between patients with an HPV-positive versus HPV-negative tumor, linear, and logistic mixed models were used with fixed effects for group (HPV-positive or HPV-negative) and measurement (pretreatment and at 6 weeks and 6, 12, 18, and 24 months after treatment) and their two-way interaction, and a random effect for subject (patients with an OPSCC tumor). To investigate potential confounding (sociodemographic (age, sex), clinical (comorbidity, tumor sublocation, tumor stage, treatment modality), lifestyle (smoking and alcohol use)) variables, these variables were included one by one in the analyses. Variables associated with HPV status and HRQOL that changed the regression coefficient of HPV status with more than 10% were included in the final model (starting with the strongest confounder) [30]. Statistical analyses were performed in SPSS 22.0 for Windows. A *p* value of 0.05 or less was considered statistically significant and indicates a significant different trajectory of quality of life over time for HPV-positive versus HPV-negative patients.

## Results

### Study sample

**Fig. 1:**
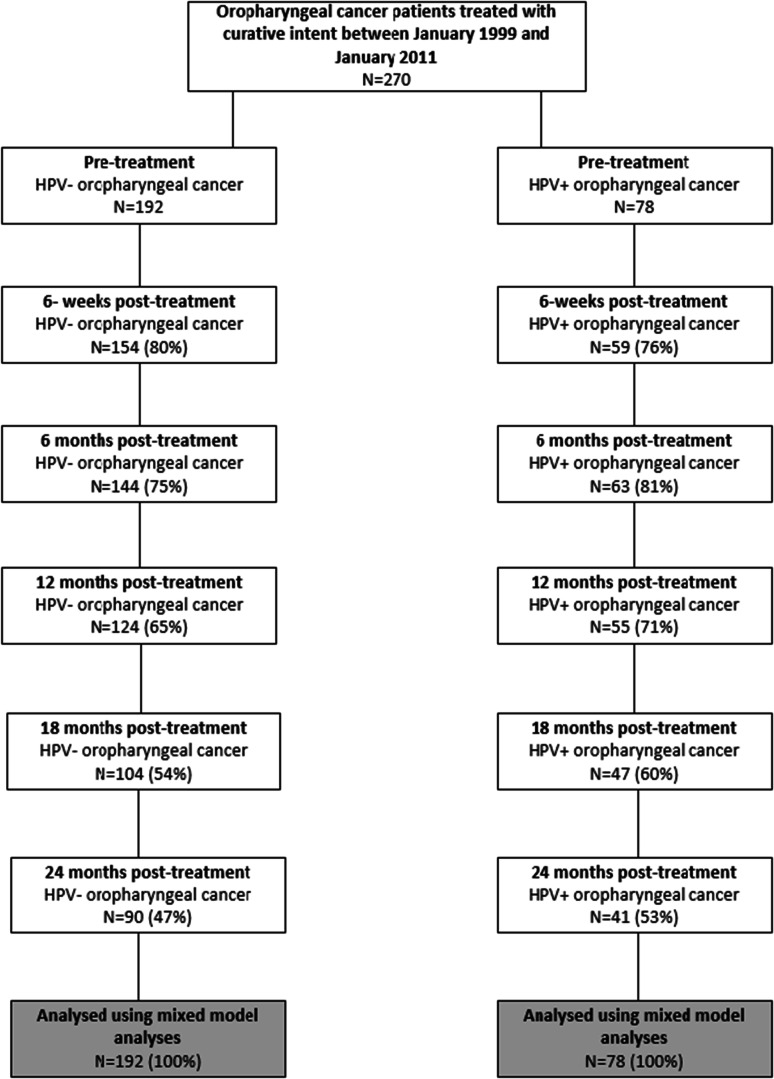
Flow diagram

In total, 270 patients met the in- and exclusion criteria, of whom 29% had an HPV-positive tumor (78/270) (Fig. 1). Patient characteristics are shown in Table 1. There were significant differences, pretreatment, between patients with HPV-positive versus HPV-negative tumors with respect to sex, comorbidity, treatment modality, smoking, and alcohol consumption. Patients with an HPV-positive tumor were significantly more often male, had fewer comorbidities, were more often diagnosed with a tumor of the tonsil or base of the tongue, were more often treated with single treatment, were less often current smokers and had lower alcohol consumption.

**Table 1:** Overview of patient characteristics (*n* = 270) in relation to HPV-status of the tumor

		HPV-status				*p* value
		Negative *N* = 192	% (71.1)	Positive *N* = 78	% (28.9)	
Age, years Mean age (SD)		59.9 (8.7)		59.9 (8.2)		0.95
Sex	Male	120	62.5%	59	75.6%	0.038
	Female	72	37.5%	19	24.4%	
Comorbidity	None	65	33.9%	42	53.8%	0.026
	Mild	59	30.7%	17	21.8%	
	Moderate	54	28.1%	15	19.2%	
	Severe	14	7.3%	4	5.1%	
Smoking	No smoking	17	8.9%	33	42.9%	< 0.001
	1–24 pack years	24	12.6%	18	23.4%	
	> 24 pack years	150	78.5%	26	33.8%	
Alcohol use	No alcohol use	19	10.0%	24	31.2%	< 0.001
	1–149 units per year	41	21.6%	29	37.7%	
	> 149 units per year	130	68.4%	24	31.2%	
Tumor location	Base of tongue	44	22.9%	23	29.5%	< 0.001
	Soft palate	37	19.3%	4	5.1%	
	Tonsil	74	38.5%	48	61.5%	
	Oropharynx nos	37	19.3%	3	3.8%	
TNM classification	Stage I	27	14.1%	10	12.8%	0.24
	Stage II	47	24.5%	14	17.9%	
	Stage III	45	23.4%	14	17.9%	
	Stage IV A–B	73	38%	40	51.3%	
Treatment	Single treatment*	103	53.6%	25	32.1%	0.001
	Combination treatment	89	46.4%	53	67.9%	

*HPV* human papilloma virus; *SD* standard deviation; *nos* not otherwise specified

*This comprised RT: *n* = 99, surg *n* = 4

### The course of HRQOL in relation to HPV status

The course of the HRQOL scores (mean and standard deviation) from pre-treatment to 24 months follow-up on the various subscales of the EORTC QLQ-C30 and QLQ-H&N35 is presented in Table 2.

**Table 2:** Overview of the course of the EORTC QLQ-C30 scales

		Before treatment			6 weeks after treatment			6 months after treatment			12 months after treatment			18 months after treatment			24 months after treatment			HPV × time	
																				*p* value	*p* value
		*n*	m	SD	*n*	m	SD	*n*	m	SD	*n*	m	SD	*n*	m	SD	*n*	m	SD	Unadjusted	Adjusted for
**QLQ-C30**																					
Global quality of life	HPV-negative	186	64.3	21.2	153	62.1	22.2	139	66.4	22.1	121	67.5	23.8	101	71.0	22.6	88	71.3	21.4	0.054	0.038
	HPV-positive	78	70.1	23.2	58	62.9	19.1	62	71.2	21.5	53	78.3	19.6	46	80.1	18.0	40	82.3	15.7		Tumor stage
Physical functioning	HPV-negative	182	79.9	20.3	148	71.2	22.8	137	76.9	20.9	122	75.7	20.9	100	78.1	22.2	87	80.6	21.2	<0.001	< 0.001
	HPV-positive	75	85.1	20.8	54	69.9	22.8	63	81.7	19.8	53	85.9	20.8	46	88.0	15.9	40	91.3	9.8		
Role functioning	HPV-negative	183	69.9	34.3	150	62.2	32.0	142	73.0	29.5	119	71.7	30.5	103	77.8	28.3	86	79.8	26.5	0.006	0.004
	HPV-positive	77	78.0	30.0	56	55.7	30.7	62	72.8	27.9	55	83.0	27.3	45	90.0	15.7	41	91.1	15.9		Tumor stage + sex
Emotional functioning	HPV-negative	181	64.6	22.1	154	73.3	25.5	140	76.4	23.9	123	75.7	25.8	95	79.6	23.5	88	83.0	23.1	0.039	
	HPV-positive	76	65.9	21.9	57	75.0	21.4	62	77.3	23.9	53	85.1	19.9	46	86.6	19.2	37	93.2	11.6		
Cognitive functioning	HPV-negative	187	82.8	23.3	153	82.9	22.0	140	86.0	18.5	122	82.7	22.4	101	83.0	21.7	87	87.7	16.4	0.14	
	HPV-positive	77	82.7	22.9	58	79.6	21.6	62	83.6	17.5	53	88.4	16.5	47	88.3	13.9	39	89.3	13.5		
Social functioning	HPV-negative	187	80.5	25.4	151	76.3	25.3	139	80.5	23.6	122	80.5	25.0	100	80.8	27.7	88	86.9	19.2	0.033	0.021
	HPV-positive	76	82.0	25.1	58	73.3	24.2	61	82.8	21.7	52	88.5	19.7	46	89.5	20.6	39	94.0	11.8		Tumor stage
Fatigue	HPV-negative	186	34.2	27.1	153	43.3	28.0	140	34.0	25.4	120	36.7	27.0	99	29.2	26.7	86	24.4	24.7	<0.001	< 0.001
	HPV-positive	76	27.3	25.2	57	46.2	28.7	62	31.5	23.2	53	18.7	22.1	46	19.3	20.3	40	13.1	15.3		Sex
Nausea and vomiting	HPV-negative	189	8.0	18.0	154	14.9	23.8	142	9.3	19.1	121	8.7	20.9	100	7.2	17.4	88	4.9	13.6	0.34	0.32
	HPV-positive	78	3.6	11.3	59	16.1	21.7	62	6.2	13.2	53	4.4	13.5	47	1.4	4.7	40	1.3	4.4		Sex
Pain	HPV-negative	187	31.9	27.9	153	32.1	28.7	141	24.3	27.6	122	24.2	30.9	100	22.9	30.8	88	15.2	20.6	0.042	0.039
	HPV-positive	77	19.9	22.9	58	29.6	25.9	62	20.7	25.6	53	12.6	17.9	46	9.4	16.7	39	8.1	17.5		Sex
Dyspnoea	HPV-negative	192	20.5	28.1	153	18.1	25.9	144	15.5	24.3	124	18.3	26.0	103	15.2	25.9	89	15.0	23.6	0.64	0.64
	HPV-positive	77	14.7	22.6	59	15.8	22.6	63	14.3	23.7	55	10.3	22.1	47	14.2	26.7	41	8.1	16.3		Comorbidity
Insomnia	HPV-negative	192	37.2	32.1	154	29.9	32.6	144	27.8	32.3	124	27.4	34.0	104	22.8	33.6	90	23.0	32.2	0.046	0.034
	HPV-positive	78	27.8	31.1	59	33.3	29.7	62	24.7	30.1	55	15.2	23.0	47	17.0	24.9	40	10.0	18.8		Sex + tumor stage
Appetite loss	HPV-negative	190	21.8	26.0	148	34.5	36.1	141	27.0	32.8	121	23.7	29.6	100	16.3	25.7	84	18.7	27.1	0.005	
	HPV-positive	77	13.0	24.9	54	43.8	33.5	62	25.3	32.3	54	11.7	25.2	46	7.2	18.5	41	7.3	19.0		
Constipation	HPV-negative	188	8.7	18.0	154	24.7	31.8	141	10.2	22.9	122	12.6	26.5	101	7.9	18.9	88	10.2	21.7	0.47	0.45
	HPV-positive	78	6.8	17.3	59	21.5	28.2	62	11.8	21.8	53	8.2	15.9	47	7.1	16.9	40	4.2	17.2		Sex
Diarrhea	HPV-negative	188	11.3	21.3	153	10.2	20.0	141	6.9	16.7	122	12.0	25.0	101	10.3	21.5	88	9.1	20.7	0.37	
	HPV-positive	78	6.8	17.3	59	6.8	16.1	62	5.4	13.8	53	3.1	9.8	47	3.5	10.4	40	0	0		
Financial Difficulties	HPV-negative	187	12.0	24.8	153	13.1	24.8	142	13.6	27.8	122	10.4	22.7	100	14.3	26.9	89	9.4	20.7	0.50	0.48
	HPV-positive	77	9.5	17.0	59	10.7	20.9	61	6.6	18.1	53	10.1	21.3	47	10.6	22.1	40	4.2	11.2		Tumor stage

*m* mean; *SD* standard deviation

Scores are based on a scale of 0–100, with a higher score suggesting a higher level of functioning or higher level of symptoms or problems

Multivariate mixed model analyses revealed that, after correcting for confounders (sex, comorbidity, tumor stage, treatment modality), the course of HRQOL was significantly different between patients with an HPV-positive tumor versus patients with an HPV-negative tumor, regarding global quality of life physical functioning, role functioning, emotional functioning, social functioning, fatigue, pain, insomnia, appetite loss (EORTC QLQ-C30, Table 3), and oral pain (EORTC QLQ-H&N35) (Table 4).

**Table 3:** Overview of the course of the EORTC QLQ-H&N35 scales

		Before treatment			6 weeks after treatment			6 months after treatment			12 months after treatment			18 months after treatment			24 months after treatment		
		*n*	m	SD	*n*	m	SD	*n*	m	SD	*n*	m	SD	*n*	m	SD	*n*	m	SD
QLQ-H&N35																			
Oral pain	HPV-negative	186	37.0	27.7	150	34.7	29.7	141	32.0	26.7	122	32.9	26.4	98	34.2	28.1	86	28.0	24.8
	HPV-positive	75	22.3	23.8	57	24.9	21.0	60	31.4	26.5	53	23.3	23.2	46	22.1	23.4	40	25.2	23.5
Swallowing	HPV-negative	162	27.8	24.6	136	34.9	29.8	129	31.1	26.9	104	30.3	25.9	90	33.1	27.2	82	30.0	28.5
	HPV-positive	72	21.8	25.7	55	29.5	27.3	56	35.1	29.9	46	27.2	28.7	41	25.6	25.9	37	22.7	21.1
Senses	HPV-negative	184	18.7	24.4	146	22.7	27.5	142	24.3	27.8	116	26.3	28.0	101	21.6	27.6	89	24.0	26.0
	HPV-positive	77	16.5	24.7	57	22.2	25.5	59	22.3	26.0	51	17.2	21.7	45	19.3	23.8	41	16.7	22.0
Speech	HPV-negative	182	18.3	20.7	145	22.3	24.5	139	19.0	22.2	117	19.5	20.1	97	20.5	22.9	86	17.8	22.7
	HPV-positive	76	12.3	20.0	58	16.7	24.0	61	19.9	23.4	54	14.8	18.3	47	16.3	22.2	41	11.4	17.8
Social eating	HPV-negative	168	23.9	24.2	139	30.8	29.6	126	30.8	26.5	101	32.3	27.0	87	30.3	25.9	77	31.0	29.7
	HPV-positive	72	21.5	28.4	54	28.9	27.7	53	28.3	28.4	46	23.2	28.7	43	24.8	26.1	38	18.0	23.6
Social contact	HPV-negative	172	7.4	12.0	140	11.6	17.9	134	12.4	19.6	108	11.5	18.4	95	10.2	15.0	82	9.7	16.5
	HPV-positive	72	5.5	15.0	56	9.4	18.7	60	10.3	19.5	52	4.6	11.2	47	5.5	15.8	39	3.8	6.1
Sexuality	HPV-negative	148	31.9	35.7	117	34.3	37.7	104	27.6	35.4	94	31.2	32.1	78	30.8	35.6	63	34.1	35.8
	HPV-positive	65	23.6	33.8	49	32.3	34.8	56	31.0	35.7	48	28.5	35.4	40	25.4	32.9	39	21.0	28.5
Problems with teeth	HPV-negative	178	22.8	32.3	138	27.5	36.0	133	26.3	34.8	107	21.5	28.7	93	22.6	31.5	87	20.3	29.8
	HPV-positive	76	14.0	27.9	55	14.5	27.0	60	15.6	29.1	51	15.0	26.1	46	14.5	25.0	40	13.3	27.0
Problems opening mouth	HPV-negative	188	34.6	34.9	152	36.4	36.7	142	35.9	35.7	122	41.3	34.8	102	35.0	37.0	90	35.9	35.8
	HPV-positive	78	27.8	32.9	58	29.9	35.7	61	37.2	35.0	53	29.6	33.8	47	29.1	35.2	41	26.8	31.0
Dry mouth	HPV-negative	190	45.4	36.4	152	51.5	36.8	142	53.8	37.2	120	53.3	35.7	102	50.3	36.0	89	51.7	40.8
	HPV-positive	78	43.2	36.5	58	62.1	33.3	61	60.7	51.1	54	50.0	37.1	47	48.9	36.7	41	41.5	28.7
Sticky saliva	HPV-negative	188	41.8	33.4	150	48.9	37.2	137	49.6	64.6	118	50.8	35.3	98	46.9	35.8	85	43.5	35.6
	HPV-positive	78	35.5	35.0	55	49.1	35.1	61	41.0	35.2	51	40.5	37.3	46	39.1	35.3	40	36.7	32.7
Coughed	HPV-negative	189	25.4	27.5	152	29.2	27.7	142	26.5	26.5	122	30.6	29.6	101	26.4	30.3	90	23.7	27.0
	HPV-positive	77	19.5	26.7	58	28.7	25.3	61	29.0	26.2	54	22.8	29.5	47	22.7	23.2	41	23.6	30.0
Feeling ill	HPV-negative	189	17.8	25.6	152	20.0	28.5	142	16.4	26.6	121	19.8	29.7	102	19.0	29.9	90	16.3	27.5
	HPV-positive	77	12.6	24.8	58	13.2	19.7	61	18.0	26.2	54	13.6	25.5	47	9.9	19.6	41	14.6	26.9
Painkillers	HPV-negative	187	50.8	50.1	153	50.3	50.2	144	53.5	50.1	123	56.1	49.8	103	46.6	50.1	87	51.7	50.3
	HPV-positive	75	33.3	47.5	58	36.2	48.5	61	44.3	50.1	54	38.9	49.2	47	36.2	48.6	41	46.3	50.5
Nutritional supplements	HPV-negative	185	44.3	49.8	148	48.0	50.1	143	53.2	50.1	119	51.3	50.2	102	50.0	50.2	87	42.5	49.7
	HPV-positive	74	36.5	48.5	58	39.7	49.3	62	40.3	49.5	54	29.6	46.1	46	32.4	47.4	41	26.8	44.9
Feeding tube	HPV-negative	185	19.4	39.7	150	24.0	42.9	144	18.1	38.6	123	19.5	39.8	103	18.5	39.0	87	16.1	37.0
	HPV-positive	75	14.7	35.6	58	22.4	42.1	62	24.2	43.2	53	28.3	45.5	47	14.9	36.0	41	9.8	30.0
Lost weight	HPV-negative	184	31.0	46.4	151	37.8	48.6	142	38.7	53.1	123	29.3	45.7	100	37.0	48.5	87	33.3	47.4
	HPV-positive	73	21.9	41.7	58	22.4	42.1	61	32.8	47.3	53	20.8	40.9	47	17.0	38.0	41	14.6	35.8
Gained weight	HPV-negative	182	29.7	45.8	149	32.2	46.9	140	27.9	45.0	122	31.2	46.5	101	31.7	46.8	86	29.1	45.7
	HPV-positive	72	31.9	47.0	58	31.0	46.7	60	26.7	44.6	53	30.2	46.3	47	29.8	46.2	41	22.0	41.9

*m* mean; *SD* standard deviation

Scores are based on a scale of 0–100, with a higher score suggesting a higher level of symptoms or problems

**Table 4:** Results of mixed model analyses for all patients regarding the course of head and neck cancer-specific Quality of Life (EORTC QLQ-H&N35) over time

	HPV × time *p* value unadjusted	*p* value adjusted
**Pain** sex + alcohol use	0.017	0.018
**Swallowing** tumor stage	0.20	0.19
**Senses** -	0.86	
**Speech** tumor stage	0.42	0.42
**Social eating** tumor stage	0.70	0.61
**Social contact** tumor stage	0.37	0.31
**Sexuality** tumor stage + comorbidity	0.43	0.38
**Problems with teeth** tumor stage	0.89	0.92
**Problems opening mouth** treatment + tumor stage	0.61	0.60
**Dry mouth** sex + tumor stage	0.19	0.15
**Sticky saliva** tumor stage	0.93	0.88
**Coughed** tumor stage	0.34	0.33
**Felt ill** tumor stage	0.30	0.27
**Painkillers** sex + alcohol use + tumor stage	0.83	0.94
**Nutritional supplements** tumor stage	0.76	0.65
**Feeding tube** tumor stage	0.30	0.30
**Lost weight** -	0.46	
**Gained weight** -	0.97	

**Fig. 2:**
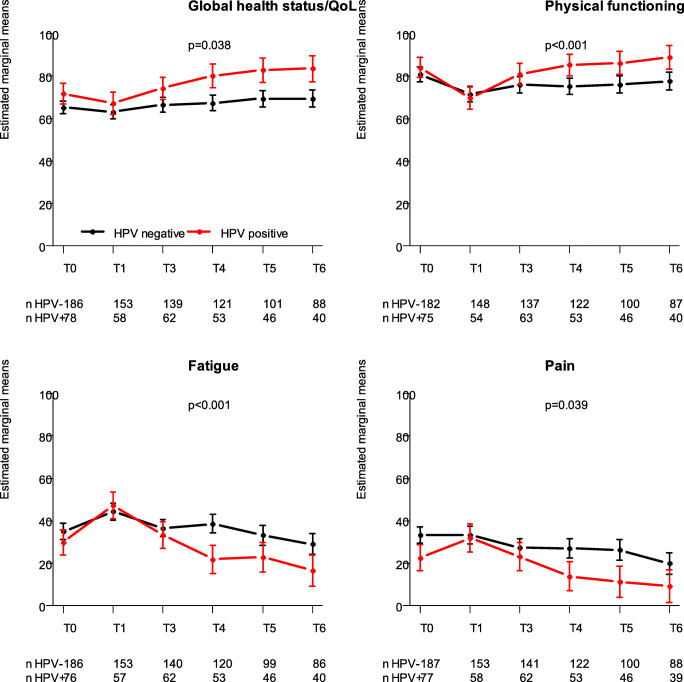
Change over time for HPV-positive versus HPV-negative patients. Estimated marginal means—corrected

Three trajectories were observed (unadjusted). With respect to global quality of life, physical functioning, role functioning, social functioning, fatigue, pain, insomnia, and appetite loss, patients with an HPV-positive tumor scored equal or better before treatment, worsened more during treatment, and recovered better and faster at follow-up compared to patients with an HPV-negative tumor (Fig. 2). A second trajectory was seen for emotional functioning: mean scores were equal at baseline, and at 6 weeks after treatment and 3 months follow-up, but scores improved more in patients treated for an HPV-positive tumor at 6, 12, and 24 months follow-up compared to those treated for an HPV-negative tumor. A third trajectory was observed for oral pain (EORTC QLQ-H&N35). In patients with an HPV-positive tumor (compared to patients with an HPV-negative tumor) mean oral pain score was lower before treatment and 6 weeks after treatment, was similar at 3 months follow-up, lower again at 6 and 12 months follow-up, and similar at 24 months follow-up.

## Discussion

In this study, we found significant differences in the course of HRQOL from diagnosis to 2 years follow-up between OPSCC patients with an HPV-positive tumor compared to those with an HPV-negative tumor, when adjusted for confounders as sex, comorbidity, tumor sublocation, tumor stage, and treatment modality. These differences concerned the course of global quality of life, physical, emotional, social, and role functioning, and the symptoms fatigue, pain, insomnia, and appetite loss (EORTC QLQ-C30 subscales) and oral pain (EORTC QLQ-H&N35 subscale). No differences between both groups were found for the course of cognitive functioning, nausea/vomiting, dyspnea, constipation, diarrhea, financial difficulties (EORTC QLQ-C30 subscales), and, except for oral pain, all other HNC-specific symptoms (EORTC QLQ-H&N35 subscales).

Of the ten studies that investigated HPV status in relation to HRQOL among HNSCC patients [14–23], six investigated the association between HPV status and HRQOL before treatment [14, 17, 19, 21–23]. Two studies reported that patients diagnosed with an HPV-positive OPSCC had better HRQOL compared to patients diagnosed with an HPV-negative tumor [19, 21]. These two studies did not adjust for possible sociodemographic, clinical, or lifestyle confounders. Three other studies showed no differences between the two groups in HRQOL before treatment, when adjusting for possible confounders in multivariate analyses [14, 17, 22]. One study focusing on fatigue reported that patients with an HPV-positive OPSCC had less fatigue at baseline, when adjusting for confounders.

All ten studies investigated the association between HPV status and HRQOL during or after treatment or at follow-up. One study reported worse HRQOL in the two last weeks of treatment in patients with an HPV-positive tumor compared to those treated for an HPV-negative tumor [21]. Several studies reported better HRQOL outcome at 2–3 months [19], 6 months [19], 12 months [17, 19, 21], and beyond 1-year follow-up [16, 17, 19, 21] in patients treated for an HPV-positive tumor compared to those treated for an HPV-negative tumor. In contrast, several other studies reported no significant differences in HRQOL between the two groups posttreatment [20], at 2–3 months [17], 6 months [17], 12 months [15], or beyond 1-year follow-up [18]. In all these analyses, possible sociodemographic, clinical, or lifestyle confounders were not adjusted for. Three studies did adjust for possible confounders [14, 22, 23]. A study among 112 OPSCC patients treated with free-flap reconstructive surgery reported that speech intelligibility and swallowing safety do not seem to differ between both groups at baseline or 6 or 12 months follow-up, when adjusted for age, sex, and tumor stage [22]. The same holds for HRQOL, except for social functioning which was better at follow-up in patients treated with an HPV-positive tumor [22]. Sharma et al. [14] reported similar findings on the course of the overall score of the HNC-specific University of Washington Quality of Life questionnaire from baseline to 1-year follow-up: patients with an HPV-positive tumor scored better before treatment, worsened much more during treatment, and recovered better and faster at follow-up compared to patients with an HPV-negative tumor. However, when they adjusted for possible confounding factors (age, sex, race, tumor subsite and stage, treatment modality, comorbidity, smoking, alcohol use), this difference was no longer significant. This may be explained by the fact that they used an overall score of the HNC-specific HRQOL questionnaire, which matches the findings in the present study that there were no significant differences between the two groups on the course of nearly all HNC symptoms (except oral pain). The course of oral pain in the present study was also significantly different between the groups but with a different trajectory: patients with HPV-positive OPSCC reported less oral pain before treatment and 6 weeks after treatment, similar scores at 3 months follow-up, less again at 6 and 12 months follow-up, and similar at 24 months follow-up, as compared to patients with HPV-negative OPSCC. This pattern is hard to understand. It may be a statistical anomaly although the differences were also clinically relevant (difference of > 10 points). It may also be caused by the nature of the EORTC QLQ-H&N35 oral pain subscale which consists of 4 items related to pain in the mouth, pain in the jaw, soreness in the mouth, and a painful throat. More research on the association between HPV status and pain is needed. The third study that adjusted for confounders reported that in patients with an HPV-positive tumor fatigue increased more from baseline to 1-month follow-up but recovered better at 3 months follow-up compared to patients with an HPV-negative tumor [23].

In the “Introduction” section, based on these previous studies [14–23], we suggested that the course of HRQOL of patients with an HPV-positive tumor (compared to those diagnosed with an HPV-negative tumor) may be better before treatment, deteriorates much more during treatment, but recovers better from 3-month follow-up and beyond. In the current study, we showed that these different trajectories are present regarding physical, social, and role functioning, and the symptoms fatigue, pain, insomnia, and appetite loss, also when adjusted for sociodemographic, clinical, and lifestyle confounders.

Differences in the course of HRQOL between patients with an HPV-positive versus HPV-negative OPSCC, especially the deterioration shortly after treatment in HPV-positive patients, may be explained by acute toxicity. Ringash et al. [17] examined the rate of acute toxicity at 2 months after treatment by HPV status. Patients with HPV-positive tumors had a higher frequency of grade 2 to 4 toxicity for nausea, vomiting, and salivary changes. Although in the present study, the course of nausea and vomiting, sticky saliva, and xerostomia from baseline to 2 years follow-up was not significantly different, patients with HPV-positive tumors had worse scores regarding xerostomia at 6 weeks and 3 months follow-up. Ringash et al. [17] also found that asymptomatic neutrophil and Hb toxicities at 2 months follow-up were more frequent in patients with an HPV-negative tumor compared to those treated for an HPV-positive tumor. Also Fang et al. [23] reported that fatigue increased more from baseline to 1 month after radiotherapy but was significantly less at 3 months in patients with an HPV-positive tumor compared to those with an HPV-negative tumor, and this pattern was significantly associated with inflammation as measured with plasma sTNFR2. This may explain the findings in the present study that patients treated for an HPV-positive tumor in general recover better and faster from symptoms such as fatigue, pain, and insomnia, and that physical, role, social, and emotional functioning is better in the longer term, compared to patients treated for an HPV-negative tumor. However, more research is needed to unravel these complex associations.

Strength of this study was the study design with general cancer as well as HNC-specific HRQOL assessments from baseline up to 2 years after treatment among a large sample of patients with OPSCC only. Also, HPV status was tested based on p16 as well as HPV-DNA, in contrast to previous studies that used p16 as surrogate marker of HPV status only. In addition, several sociodemographic and clinical confounders were taken into account. We also investigated the role of possible lifestyle confounders (smoking and alcohol use before start of treatment), but this did not change the outcome and was not relevant to the significance of the results. Further research is, however, warranted on the potential confounding role of continued smoking and alcohol use during follow-up, as well as other potential sociodemographic confounders such as education and living situation. These factors were not included in our study, which should be seen as a limitation. Another limitation of this study is that we excluded patients of whom HPV status was not known and those patients without pretreatment HRQOL data, which may have induced selection bias. Furthermore, our data represent a small proportion (estimated on 10–15%) of the total number of OPSCC patients in the Netherlands. Due to the study design and privacy regulations, it was not possible to compare responders and nonresponders of this study. Another limitation is that the QOL data was acquired as part of routine clinical care and therefore depended on timing of the follow-up visits and was therefore not exactly at 6 weeks, 3, 6, 12, and 24 months follow-up. As a comparable time, window was used for both HPV+ and HPV− OPSCC patients, we, however, expect that this does not have influenced our findings. Another limitation is the existence of missing surveys due to death and, in minority, to loss to follow-up. Finally, a potential limitation of this study is that HRQQOL measured at the end of treatment was not taken into account while acute toxicities such as fatigue and appetite loss may be highest at this time point.

The findings in this study may contribute to the current discussion about treatment deescalation. Efforts to improve treatment outcome in this patient group should also focus on preventing HRQOL deterioration during and shortly after treatment when possible [31–34]. Furthermore, the results of this study are helpful in further advancing supportive care by providing information on the impact of OPSCC and its treatment on HRQOL and referring patients to supportive care, tailored to the needs of OPSCC patients with HPV-positive and those with HPV-negative tumors.

## Conclusion

Among OPSCC patients, the trajectory of the course of HRQOL is significantly different in patients with an HPV-positive versus an HPV-negative tumor, when adjusted for sociodemographic and clinical confounders. Regarding global quality of life, physical functioning, role functioning, social functioning, fatigue, pain, insomnia, and appetite loss, patients with HPV-positive OPSCC score better before treatment, deteriorate more during and shortly after treatment, and recover better and faster at follow-up, compared to patients with HPV-negative OPSCC. Furthermore the course of emotional functioning and oral pain is significantly different between the two groups, but with other trajectories.

## Data Availability

The authors have full control of the data, and data is available upon request.
